# Candidate Genes and Genetic Architecture of Symbiotic and Agronomic Traits Revealed by Whole-Genome, Sequence-Based Association Genetics in *Medicago truncatula*

**DOI:** 10.1371/journal.pone.0065688

**Published:** 2013-05-31

**Authors:** John Stanton-Geddes, Timothy Paape, Brendan Epstein, Roman Briskine, Jeremy Yoder, Joann Mudge, Arvind K. Bharti, Andrew D. Farmer, Peng Zhou, Roxanne Denny, Gregory D. May, Stephanie Erlandson, Mohammed Yakub, Masayuki Sugawara, Michael J. Sadowsky, Nevin D. Young, Peter Tiffin

**Affiliations:** 1 Department of Plant Biology, University of Minnesota, Saint Paul, Minnesota, United States of America; 2 Department of Computer Science and Engineering, University of Minnesota, Minneapolis, Minnesota, United States of America; 3 National Center for Genome Resources, Santa Fe, New Mexico, United States of America; 4 Department of Plant Pathology, University of Minnesota, Saint Paul, Minnesota, United States of America; 5 Department of Soil, Water, and Climate, and BioTechnology Institute, University of Minnesota, St. Paul, Minnesota, United States of America; University of Guelph, Canada

## Abstract

Genome-wide association study (GWAS) has revolutionized the search for the genetic basis of complex traits. To date, GWAS have generally relied on relatively sparse sampling of nucleotide diversity, which is likely to bias results by preferentially sampling high-frequency SNPs not in complete linkage disequilibrium (LD) with causative SNPs. To avoid these limitations we conducted GWAS with >6 million SNPs identified by sequencing the genomes of 226 accessions of the model legume *Medicago truncatula*. We used these data to identify candidate genes and the genetic architecture underlying phenotypic variation in plant height, trichome density, flowering time, and nodulation. The characteristics of candidate SNPs differed among traits, with candidates for flowering time and trichome density in distinct clusters of high linkage disequilibrium (LD) and the minor allele frequencies (MAF) of candidates underlying variation in flowering time and height significantly greater than MAF of candidates underlying variation in other traits. Candidate SNPs tagged several characterized genes including nodulation related genes *SERK2*, *MtnodGRP3*, *MtMMPL1*, *NFP*, *CaML3*, *MtnodGRP3A* and flowering time gene *MtFD* as well as uncharacterized genes that become candidates for further molecular characterization. By comparing sequence-based candidates to candidates identified by *in silico* 250K SNP arrays, we provide an empirical example of how reliance on even high-density reduced representation genomic makers can bias GWAS results. Depending on the trait, only 30–70% of the top 20 *in silico* array candidates were within 1 kb of sequence-based candidates. Moreover, the sequence-based candidates tagged by array candidates were heavily biased towards common variants; these comparisons underscore the need for caution when interpreting results from GWAS conducted with sparsely covered genomes.

## Introduction

Legumes are a highly diverse plant family that contains many economically important species including soybean, peanuts, and alfalfa. Legumes are especially important because they host rhizobial symbionts, which when growing in symbiosis inside of root organs called nodules, convert atmospheric nitrogen (N) into plant usable forms. This symbiosis annually contributes >50 million metric tons of N to managed ecosystems and twice this amount to natural ecosystems [1], [2]. The biochemical and genetic basis of this interaction has been the subject of decades of research and mutational screens have identified many genes involved in the establishment and maintenance of nodules and nitrogen fixation [3]. In many of these studies, *Medicago truncatula*, a diploid self-fertilizing species with a sequenced reference genome [4] has played a central role as a plant model [5]. The genomic resources available for *Medicago truncatula* have also made this a valuable model system for investigating the genetic basis of agronomic traits in legumes and other crop species [5]. To further the development of *M. truncatula* as a model for investigating the genetics of complex traits, including legume-rhizobia symbiosis, we developed resources for conducting genome-wide association study (GWAS).

GWAS, like traditional bi-parental quantitative trait locus (QTL) mapping, aims to identify genes responsible for naturally occurring phenotypic variation but allows for screening a much larger panel of accessions and, because of ancestral recombination, mapping candidate genes to a much finer scale than is possible in traditional QTL mapping [6]. While gene discovery is an important goal of GWAS, association analyses also provide a valuable tool for investigating the genetic architecture of complex traits. Although GWAS has, to date, been conducted primarily with reduced representation sampling of genomic diversity (e.g. SNP arrays) such sampling is expected to be biased against the detection of low frequency variants [7], miss SNPs that are not in high linkage disequilibrium with causative SNPs [8], [9], falsely identify SNPs because they define the genetic background on which causative SNPs are present (i.e. synthetic associations) [10], and limit the scale to which putative causative SNPs can be mapped. To avoid these limitations we sequenced a diverse sample of 226 *M. truncatula* accessions to ∼8X mean coverage (Figure S1). After aligning sequence reads to the *M. truncatula* reference genome [4] we identified 6,344,526 bi-allelic SNPs that were assayed in >100 accessions with minor allele frequency (MAF) >0.02 (MAF refers to the frequency of the SNP allele that is present in fewer that 0.5 of accessions). These 6.3 million SNPs provide an average of 1 SNP every 43 bases, considerably shorter than the average distance over which LD decays [11].

Our primary goals in this work were to *i*) explore the genetic architecture of complex traits by investigating the proportion of among accession variance that can be explained by candidate SNPs and the relationships between minor allele frequency and effect size, *ii*) identify candidate genes underlying important developmental and symbiosis traits, and *iii*) empirically explore bias associated with conducting GWAS with reduced-representation SNP arrays relative to sequence data.

## Results and Discussion

Phenotypic data on three developmental (height, flowering time, and trichome density) and five nodulation traits (total number of nodules and nodule number and strain occupancy in the upper and lower roots) were collected from each of 226 plant accessions grown in replicate and co-inoculated with two strains of *Sinorhizobium meliloti*. The sample of 226 accessions is much smaller than used in human GWAS studies, where sample sizes often exceed 10,000 individuals, but is larger or similar to the number of accessions used in GWAS in *A. thaliana* [12], [13], [14], *D. melanogastor* [15], *Oryza sativa* [16], and *Z. mays* [17], [18], in which phenotypic data can be collected in common environments on replicated genotypes. Because *M. truncatula* genotypes differ in the number of nodules they form with different rhizobia strains [19], [20] and rhizobia strains can vary in competitiveness (i.e. formation of nodules on young plants) [21], the root system of each plant was divided into upper and lower portions, the former portions showing early nodulation events [22]. The eight traits exhibited significant among-accession variation, ranging from 22% (strain occupancy in lower roots) to 74% (flowering) of the total variance (Table 1, Figure S2).

**Table 1:** Proportion of variance attributed to accessions and linkage disequilibrium for top 50 candidates SNPs.

trait	Among accesion/total variance	Proportion top 50 SNPs not in LD (r^2^<0.8)	Proportion top 50 SNPs not in LD (r^2^<0.3)	Linear regression r^2^ (SNPs in final model)	correlation between MAF and effect size, top 200 SNPs (P-value)
Height	0.58	0.98	0.91	0.75 (33)	−0.12 (0.10)
Flowering	0.74	0.89	0.59	0.64 (31)	−0.05 (0.48)
Trichomes	0.45	0.32	0.68	0.41 (17)	0.22 (0.002)
Nodules on upper roots	0.34	0.95	0.73	0.65 (27)	0.01 (0.93)
Nodules on lower roots	0.35	0.98	0.86	0.69 (32)	−0.21 (0.002)
Total nodules	0.38	0.99	0.94	0.74 (30)	−0.08 (0.24)
Strain occupancy in upper roots	0.24	0.98	0.92	0.67 (27)	0.10 (0.14)
Strain occupancy in lower roots	0.22	0.96	0.87	0.61 (24)	0.17 (0.01)

To identify candidate genes responsible for the among-accession variation we conducted GWAS with >6 million SNPs present at a MAF >0.02 using the mixed linear model [23], [24], [25] approach implemented in TASSEL [26]. Visual inspection of quantile-quantile (q-q) plots indicated that inclusion of a kinship-matrix (K) covariate removed the major effects of population structure and unequal relatedness among individuals that can bias GWAS (Figure S3). Preliminary analyses revealed that inclusion of additional measures of population structure, such as those obtained from STRUCTURE, had only very minor affects on the shape of the q-q plots or the top ranked SNPs and thus were not included in the final analyses. While including covariates describing kinship or population structure is important to reduce the numbers of false positives obtained in GWAS, including such covariates may also weaken the statistical power to identify genes responsible for trait variation when a phenotype covaries with relatedness [12], [13]. For our data, the first ten PCs of the K matrix explained from 6–36% (nodule number and flowering time, respectively) of variation among accessions.

### Genetic architecture

**Figure 1:**
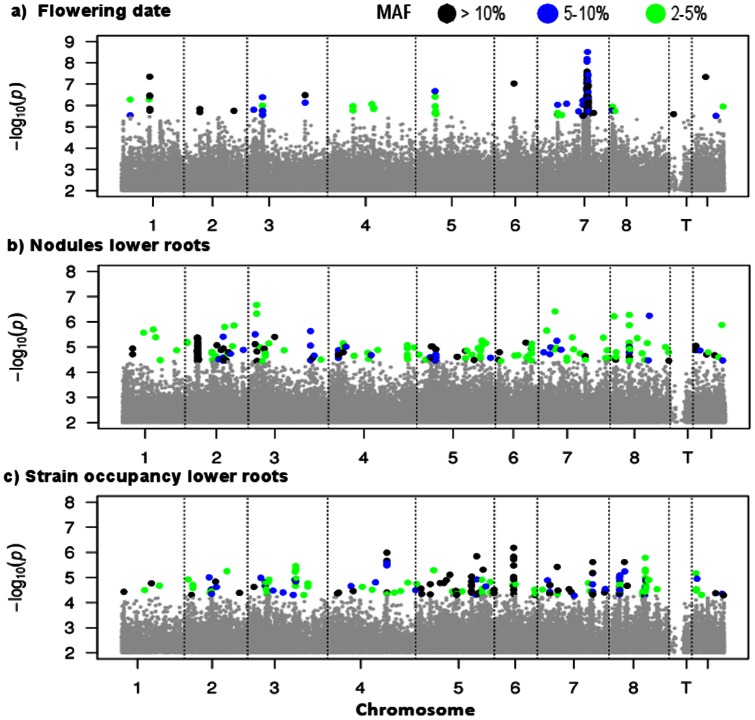
Manhattan plots showing candidate SNPs. (a) Flowering time, (b) nodules in lower roots and (c) nodule occupancy in lower roots. Colors indicate MAF of top 200 SNPs. Y-axis shows –log10(P) and X-axis is the physical location along each of the 8 chromosomes, uncaptured transcribed contigs (T), unanchored BACs (U).

For exploring genetic architecture we considered genes containing or adjacent to the 200 SNPs with the smallest *P*-values as candidates underlying phenotypic variation in each trait (Data File S1). Although this is a non-stringent criterion for identifying candidate genes and this list of candidates is therefore expected to contain false positives, the non-stringent criterion allows for the inclusion of SNPs of small effect, which would not be detected using stringent P-values but may be important contributors to complex trait variation and thus are important to consider when investigating genetic architecture. Genomic distributions of the top 200 candidate SNPs differed dramatically among traits (Figure 1, Figure S4). For most of the assayed traits candidates are spread across the genome and few candidate SNPs were in high LD with one or more other candidates (Table 1). For example, for nodule number in lower roots there are no clusters of candidate SNPs greater than 13 kb in length and only 14% of pairwise LD measures (r^2^) are >0.3. By contrast, the strongest candidates for flowering time and trichome density are in clusters of high LD. The clustering of flowering time SNPs is particularly distinct, with >75% of candidate SNPs within ∼800 kb on chromosome 7. Interestingly GWAS conducted in rice and *A. thaliana* [12], [16], [27] also identified SNPs controlling flowering time also form clusters of high LD, suggesting that few genes of fairly large effect may control a considerable amount of the variance in flowering time in each of these species. The extensive LD among flowering time and trichome density candidate SNPs also suggests that selection may maintain functionally divergent alleles, that alleles are subject to local adaptation, or have experienced recent soft-sweeps [28].

Mean MAF of candidate SNPs also differed significantly among traits (F _df = 7,1590_, P<0.0001, Table 1), ranging from 0.06 for trichome density to 0.21 for height. Candidates underlying variation in plant height had significantly greater mean MAF than either genome-wide SNPs (mean MAF  = 0.09) or candidate SNPs for other traits (all P<0.0001, Figure S5), and candidates for flowering time (MAF  = 0.16) had significantly greater MAF than all SNPs as well as candidates for all traits except height and nodule number (all P<0.01). Given that height and flowering time are likely subject to stabilizing selection, the greater MAF for these traits may reflect spatial variation in fitness optima or weak selection due to a fitness plateau near an optimum [29].

### Variance explained by top candidate SNPs

If the candidate SNPs identified through GWAS act additively and capture the majority of genomic variation for that trait, than we would expect a high proportion of the phenotypic variation in a trait be explained in a linear regression in which the candidate SNPs are used as explanatory variables. For our data, linear regression using the top 50 candidate SNPs as potential explanatory variables (17–33 SNPs retained after model simplification by AIC depending on the trait) explained 41–75% of among-genotype variance (r^2^) in phenotypes (Table 1). These values are similar to r^2^ values for GWAS candidate SNPs underlying startle response and starvation resistance in *Drosophila melanogaster* [15]. However, these values are biased because candidates used in the linear regression were pre-selected from all assayed SNPs based on their association with phenotype, i.e. the Beavis effect or winner's curse [30], [31]. In other words, even if the phenotypes are randomly associated with genotype we expect the r^2^ values of the linear regression to be greater than zero because the 50 SNPs used in the linear regression are those that GWAS identified as having the highest covariance with the phenotype of interest. For this reason, for three traits we generated approximate null expectations by conducting linear regression using SNPs identified by GWAS on data sets in which phenotypes were randomly assigned to genotypes (due to computational demands these approximate distributions are based on only 20 randomizations per trait).

Mean r^2^ values of linear regression on randomized data ranged from 0.59 for strain occupancy to 0.65 for height (Table S1). Assuming the total trait phenotypic variance that can be potentially explained by true causal SNPs is then uniformly distributed between the mean of the randomized data and one, the top 50 candidate SNPs explain 21–29% of the remaining variance for height and nodules in the lower root system. In contrast the proportion of variance explained by candidate SNPs for strain occupancy in lower roots is well within the range of values generated from the randomized datasets (Table S1). For all three traits, however, the MAF distribution of the empirical data differs from that of the randomized data – for all three traits rare alleles are underrepresented and common alleles are overrepresented in the empirical compared to randomized data – suggesting that even candidate SNPs for strain occupancy in lower roots may be biologically meaningful.

Two of the traits subject to the randomization analyses, height and nodules in lower part of roots, exhibit negative correlations between MAF and estimated effect size (i.e. the predicted phenotypic difference between SNP variants) (Table 1), similar to findings in *Drosophila melanogaster* [15]. For height the empirical correlation is less than all those from randomized data, for nodules the correlation of the top 50 SNPs is less than all randomized values and for the top 200 SNPs the empirical correlation was less than all but one of the randomized values (Table S1). Negative correlations between allele frequency and effect size are consistent with mutation selection balance models for the maintenance of genetic variation in quantitative traits [29], [32]. By contrast, the empirical correlation for strain occupancy was positive and well within the range of randomized values (Tables 1 and S1).

### Candidate genes

Many of the genes tagged by candidate SNPs have annotated functions that support a role in contributing to variation in the corresponding phenotype. For flowering time, the highest ranking (*P* = 3×10^−9^, *P*<0.05 after a conservative Bonferroni correction for multiple tests) as well as 7 other candidate SNPs are adjacent to *MtFD*, an uncharacterized gene in *Medicago* but with high sequence similarity to *A. thaliana FD* which controls expression of floral identity genes [33], [34]. In the same SNP cluster, 300 kb away from *MtFD*, lies the third highest ranking SNP (P = 9×10^−9^, *P*<0.05 after a conservative Bonferroni correction for multiple tests) within a *FAR1* homolog, a gene family containing members involved in light signaling and flowering time [35]. The cluster of flowering-time candidates identified through GWAS lies within a bi-parentally mapped QTL that contains several other genes that affect flowering time, including *CONSTANS* and *FT* homologs [36]. These genes harbor SNPs in our association panel but are not identified as candidates in our analyses. The identification of a common region in both a biparental mapping population [36] and the current GWAS study provides strong support that these candidates are not false positives resulting from population structure that is not controlled for by the inclusion of the K matrix in the linear model used to identify candidates. At the same time, colocalization of traditionally mapped QTL and GWAS candidates shows the power of sequence-based GWAS to more finely map the causative SNPs underlying biparentally-mapped QTL (although we note it is possible that variants responsible for differences between two individual lines may differ from those that can be detected in a population sample that is used in GWAS). For trichome density, the other trait that shows strong single-locus effects, a cluster of candidates (smallest P = 1.8×10^−9^, *P*<0.05 after a conservative Bonferroni correction for multiple tests) is centered at a MADS-box transcription factor, a family of genes with roles in plant development. The potential for MADS-box genes to affect trichome production has been shown in Petunia where constitutive expression of the MADS-box gene UNSHAVEN causes ectopic trichome production [37].

We find that many candidate SNPs responsible for variation in nodule traits, considering the 200 SNPs with smallest P values as candidates, are located within or near genes that forward genetics previously identified as involved in nodule formation and symbiosis (Table 2). In addition to the candidate SNPs that tagged characterized genes with known nodulation phenotypes, three strain occupancy candidates were contained within a biparentally mapped QTL for differential response to nod factors and strain specific nodule occupancy [38]. Although this QTL contains several *LysM* genes involved in nod-factor perception, the candidate SNPs we identified are neither in nor adjacent to these genes. However, the genomic structure of the QTL region from the biparental mapping population [38] and that in the *M. truncatula* reference genome [4], [38] differ, suggesting this region segregates multiple arrangements within *M. truncatula*. Such rearrangements make it possible that the candidates we identified are closer to *LysM* genes in some accessions than they are in the reference genome. Alternatively, other genes within the QTL that are tagged by candidate SNPS (LRR containing *HCR6*, *HCR7* [homologous to *Cladosporium fulvum* (*Cf*) resistance] and uncharacterized *B*, *F* genes) may contribute to differences in strain occupancy, or the GWAS candidates may tag distant regions involved in gene regulation.

**Table 2:** Characterized genes associated with candidate SNPs for nodulation traits.

Trait	Gene name	Function
*Nodules upper roots*	Calmodulin *CAML3*	signaling during nodule formation [51]
	*NFP (Nod Factor Protein)*	nod factor receptor, acts upstream of other nod signaling genes [52]
	*SERK2*	signaling during defense and development [53],
*Nodules, total & lower roots*	*MtnodGRP3A*	nodule development, nodule-specific expression induced by rhizobial infection [39]
	*chit4*	chitinase with rhizobial strain-specific expression [54]
*Total nodules*	*MtN5*	nod factor induced [55]
*Occupancy upper roots*	Calmodulin *CAML2*	signaling during nodule formation [51]
	*MCA8*	predominant ATPase functioning in symbiotic Ca_2_^+^ signaling [56]
	*MtnodGRP1B*	Nodule specific glycine rich protein, expressed primarily in young nodules, in nodule apex [39]
	*MtNRT1.3*	NO_3_^−^ dependent expression, involved in primary root growth and NO_3_^−^ sensing [57]
*Occupancy lower roots*	*MtHMGR3*	strongly expressed in nodules, binds *NORK* which controls rhizobia infection [58]
	*MtMMPL1*	nodulin with rhizobia-signal dependent expression, affects infection thread size and number of viable bacteria inside of nodules [59]

Also noteworthy is that the top two candidate SNPs underlying variation in the strain occupancy in the lower roots (P = 6.5×10^−7^, 1×10^−6^) tag uncharacterized genes with evidence for expression in the nodules and roots only. Candidates underlying variation in nodule number are also overrepresented among genes with nodule- or root-specific expression; 8 of the top 20 SNPs (40%) associated with the number of nodules in lower roots that tag expressed genes show complete nodule- or root-specific expression, by comparison only 850 of the 21,000 genes (4%) located on chromosomes 1–8 for which expression was assayed show nodule or root specific expression (P<0.001). One of these eight genes is annotated as a nodule-specific glycine rich gene, a member of a small gene family involved in nodule development [39], however, the other seven (annotated as encoding albumin, PRP, RNA-binding, a U-box containing and three hypothetical proteins) have not been previously identified as affecting nodule traits.

### Reduced representation compared to sequence-based genotyping

The vast majority of GWAS have been conducted using genomic markers that provide much sparser genome coverage than sequence data. Although full sequence data is expected to soon be available for model systems, there is considerable interest in using reduced-representation genotyping (such as genotype-by-sequencing (GBS; [40]) restriction-site associated DNA (RAD-tag; [41]) in order to conduct GWAS in non-model species. Reduced-representation genotyping is appealing because of the lower financial costs and less demanding bioinformatic analyses; however, if sparsely sampled SNPs strongly bias GWAS results such studies may be misleading with regard to identification of causative variants and genetic architecture [7], [8], [9]. To assess the extent of this potential bias we conducted 100 GWAS for three traits using *in silico* 250 K SNP-platforms. The *in silico* platforms were generated using a discovery panel of 26 accessions that had been sequenced to median mapped coverage of ∼15X [11]. From these data we selected a single SNP from each of 224,339 1-kb windows that harbored segregating sites and an additional 25,662 random SNPs to produce 250 K assayed SNPs. Given that LD in the discovery panel extends an average of ∼3 kb [11], 1 SNP kb^-1^ provides relatively high density SNPs in both physical and recombination distances.

**Figure 2:**
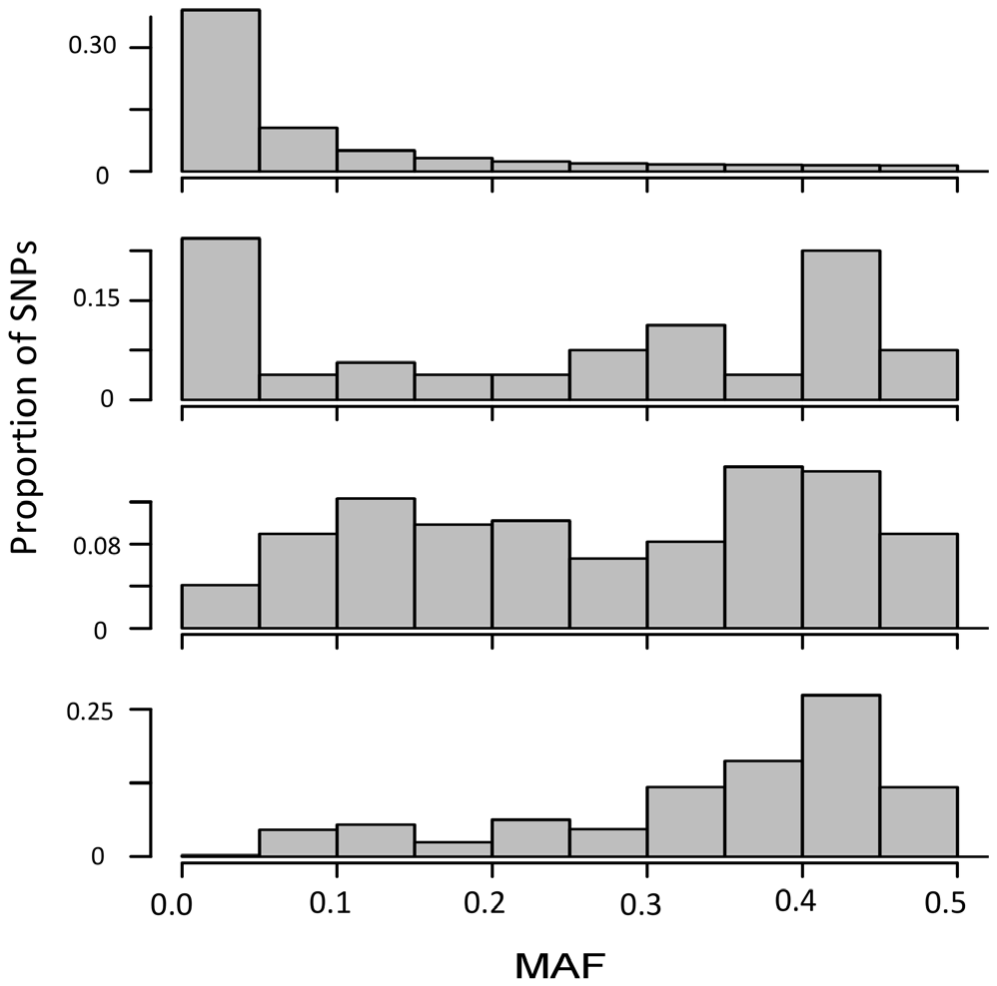
MAF distribution of genomic and candidate SNPs (minor allele frequency >0.02) identified using sequence data and 250 K SNP arrays. Shown are (a) all assayed SNPs, (b) sequence-based candidates for height, (c) top 50 candidate SNPs from 100 *in silico* platforms, and (d) distributions of sequenced based candidates within 1 kb of any of the top 50 *in silico* candidates.

The comparison of GWAS with *in silico* arrays compared to the sequence data revealed that candidates identified by the *in silico* arrays were often distant from the top sequence-based candidates and highly biased towards common variants. With regard to *in silico* candidates being located close to sequence candidates, the best performance was for height where an average of 14 of the top 20 and 19 of the top 50 *in silico* SNPs were within 1 kb of one of the top 200 sequence-based candidates, and 17 of the top 20 and 30 of the top 50 *in silico* SNPs were within 20 kb of a sequenced candidate (Table 3). By comparison, <60% of *in silico* candidates were within 20 kb of a sequence-based candidate for nodule number and occupancy in the lower roots. Not only were many *in silico* candidates not within 20 kb of sequenced-based candidates, but the tagged SNPs were not a representative sample of the empirical candidates. For all traits, there were far fewer low-MAF candidates from *in silico* than sequence data (*e.g.* 13 vs. 41% SNPs with MAF <10% for height, P<0.0001, Figure 2, Figure S6). This bias towards high-frequency candidates in the *in silico* data is expected given that *in silico* SNPs were ascertained from a 26 accession discovery panel. Moreover, because the array SNPs are unlikely to be causative, but rather identified because they are in LD with causative SNPs, the sequenced-based candidates that were tagged by *in silico* candidates are even more heavily biased towards common variants; across the three traits nearly 59% of empirical SNPs have MAF <10%, but only 15% of the platform-tagged sequenced-based candidates have MAF <10%. For height, only 5% of the platform-tagged sequence candidates had MAF <10%. Taken together, these results suggest that sequence-based GWAS is likely to provide a very different picture of the genetic architecture of complex traits than would be obtained using reduced-representation genotyping data.

**Table 3:** Overlap in candidate SNPs identified using sequence data compared to in silico SNP arrays.

	top 20	top 20	top 50	top 50
	1 kb	20 kb	1 kb	20 kb
Height	14.1(9–19)	17.2(12–20)	18.9(12–26)	30.2(22–38)
Nodule numberlower roots	9.4(4–15)	11.5(6–16)	14.0(6–21)	21.2(12–29)
Strain occupancylower roots	6.3(2–12)	9.4(4–16)	8.4(3–16)	16.5(10–25)

Shown are the average number of top 20 and 50 *in silico* candidate SNPs within 1 and 20 kb of one of the top 200 sequenced-based candidates. Data are from 100 250 K SNP *in silico* platforms, the minimum and maximum number of tagged sequence candidates is in parentheses.

### Conclusion

Genome wide association studies require high-density marker data for a large number of accessions, by conducting whole-genome sequencing and calling SNPs segregating among >250 accessions of *Medicago truncatula* this resource is now available for other researchers. In addition, seeds for the accessions that comprise the association panel we analyzed are publicly available. Together, these resources provide a valuable resource for identifying causal variants and the genomic architecture of complex traits in legumes. Our GWAS of plant height, flowering time, trichome density, and five nodule-related traits identified both uncharacterized and previously characterized genes that are likely responsible for naturally occurring variation in these traits. In addition to identifying candidate genes for functional characterization, our analyses highlight the advantages of high resolution SNP data for studying the genetic architecture of complex traits and provide an empirical example of the need for caution that should be exercised when interpreting results from GWAS conducted using sparse genotypic data.

## Materials and Methods

We sequenced 288 *Medicago* accessions (www.medicagohapmap.org/hapmap/germplasm) including the majority of lines contained in the INRA core collection [42] (www1.montpellier.inra.fr/BRC-MTR/). Prior to GWAS 62 accessions were excluded; 18 because they are highly diverged from others (Figure S7) and 44 because they were not phenotyped. Each accession was self-fertilized for ≥3 generations prior to growing for DNA extraction. Paired-end Illumina sequencing libraries (∼200–450 nt insert sizes) were prepared for sequencing according to standard methods [43] using total DNA extracted from a pool of ∼30 day-old dark-grown seedlings. Libraries were sequenced using GAII or GAIIx Illumina sequencing instruments to yield paired 90 mer or 151 mer reads (trimmed to 90 mers for analysis). Illumina image analysis pipeline with default parameters was used for base-calling, quality filtering, and to remove adapter and PhiX contamination.

Reads that passed initial quality control filtering were aligned to the *M. truncatula* reference genome v.3.5 [4] (www.medicagohapmap.org) using GSNAP [44]. After excluding reads <91% identical to a genomic region or that aligned to ≥5 locations we called SNPs when: *i*) a position was covered by ≥2 unique reads for the 26 accessions sequenced to ∼15X mapped coverage [11] (deep 26) or ≥1 unique read for other accessions, with unique reads defined as those that align to only one position in the reference genome (coverage information at www.medicagohapmap.org), and *ii*) reads that called a non-reference allele had a quality score ≥10 and variant nucleotides were called by >70% of reads. The >70% of reads calling a variant means that there are no heterozygous sites within individuals. This should have minor effects given high selfing rates in natural populations (>95%) [45], [46] and ≥3 generations of selfing prior to DNA extraction. Positions with >1000 (deep 26) or >500 unique reads for shallow accessions were excluded to prevent variant calling SNPs in repetitive regions that appear only once in the reference genome. Sequence data are available at NCBI short-read archive (SRP001874) and called SNPs for the 288 accessions are available at www.medicagohapmap.org/downloads/mt35. Because of the very high SNP density, an average of 1 SNP 50 bp^−1^ in a species in which previous analyses of genome-wide SNPs indicated LD to extend an average of 3,000 bp [11], we did not impute missing SNPs.

### Phenotype data

During February 2011, seeds from each of 226 genotypes were planted into bleach-sterilized 650 ml conetainers filled with an equal mixture of steam-sterilized Sunshine Mix LP5 (low nutrient potting soil) and Turface. Prior to planting, seeds were scarified in sulfuric acid for 5 minutes, rinsed, sterilized in 10% bleach for 90 seconds, rinsed, and cold-stratified (4°C) on sterile filter paper for 4 days. Seeds were then placed in the dark at room temperature for ∼16 hours prior to planting. After planting, one replicate from each genotype was placed in each of eight randomized complete blocks in a single greenhouse room (22°C, supplemental lighting used to maintain a 16∶8 hour light:dark cycle). Pots were adjacent to one another and plants were top-watered with a fine mist sprayer as necessary. Seeds for all accessions are available by submitting an on-line seed request form at medicagohapmap.org or by contacting INRA-Montpellier (Jean-Marie Prosperie) or the Western Regional Plant Introduction Station (WRPIS) at Washington State University directly.

Plants were inoculated two days after planting with 1 ml (∼10^7^ cells) of a nearly equal mixture of two strains of *S. meliloti*, M249 and KH46c (55% and 45%, respectively, based on plate counts), that preliminary experiments revealed to differ in nodulation phenotypes. Innocula was grown in TY medium (30 °C, ∼72 hours) then diluted 1∶200 (KH46c) or 1∶400 (M249) in 0.85% saline solution.

From the 1,899 plants that germinated and survived until harvest, we collected data on height, flowering date, trichome density, nodule number and rhizobia strain occupancy in the top 5 cm of roots (upper root) and roots below the top 5 cm (lower roots), as well as total nodule number. Plant height (length from cotyledons to tip of the farthest branch) was measured 10 weeks after planting, 1 week before plants were harvested. Time to first flower was assayed every 3–4 days starting 6 weeks after emergence. Plants that did not flower at the time of harvest (11 weeks after planting) were treated as having not flowered in analyses. For GWAS, flowering date was treated as a continuous variable with 9 flowering dates and a 10^th^ category for plants that never flowered. Though this distribution was non-normal (Fig. S2), the q-q plot was reasonable (Fig. S4) and was not improved by any transformation (not shown). Trichome density was measured as the number of trichomes visible at 10X magnification along a 2 mm section of the petiole of 1 fully expanded leaf. After harvest, roots were washed and nodules counted in the upper and lower roots. For plants from 6 blocks, ≤24 nodules (≤12 nodules from the upper root) were haphazardly sampled for strain occupancy assays using a dot-blot antibody assay [47]. In brief, nodules were dried (65°C, >48 hours), then rehydrated in 30 ul PBS, crushed, and then the supernatant was blotted onto two nitrocellulose membranes (BioRad) which were treated with one antibody each (antibodies obtained from rabbit antiserums prepared using boiled bacterial cells by Covance Inc, Denver PA). Membranes were dried and a positive antibody reaction was visually scored by a dark spot. Because anti-M249 antibody was less specific than anti-KH46c antibody (determined by control blots on every membrane), we grouped nodules into two classes: those that reacted with anti-M249 only and those that reacted with either both antibodies or anti-KH46c only. Strain occupancy data are reported as proportion of total nodules formed by strain M249. All phenotype data are available at datadryad.org dx.doi.org/10.5061/dryad.pq143.

### Genome-wide association analysis

GWAS and other analyses were conducted using the least-squares means values for each accession after removing among-block block differences. We used the efficient mixed-linear model approach expedited (EMMAX [23], P3D [25]) as implemented in TASSEL 3.0 [26] for association analyses using only SNPs scored in ≥100 accessions (median coverage  = 182 accessions) with minor allele frequency (MAF) ≥0.02.

In all analyses, we included a kinship matrix (*K*) to lessen confounding effects of population structure. The *K* matrix was calculated in TASSEL using 5,000 randomly sampled SNPs from each chromosome. Correcting for multiple testing in association analyses is problematic because of the large number of tests and dependency of P-values, as well as false discovery rates (FDR) on the distribution of the data [12], [48]. Therefore when exploring genetic architecture we considered either the 200 SNPs or 50 SNPs with lowest P-values as candidates responsible for phenotypic variation. To identify annotated candidate genes and examine expression of genes tagged by candidate SNPS we treated SNPs falling within a coding region to tag that gene and intergenic SNPs to tag the nearest adjacent gene. These analyses were conducted only for SNPs found on one of the 8 assembled chromosomes found in reference genome Mtv3.5. To identify previously characterized genes that were tagged by candidate SNPs we used BLAST to identify the Mtv3.5 genomic location of 440 named *M. truncatula* nuclear genes found in GenBank. Candidate SNPs that were within 10 kb of a named gene were considered as tagging that gene.

To estimate the proportion of phenotypic variance explained by candidate SNPs, we extracted genotype information for the top 50 SNPs and included these in a multiple linear regression with phenotype values as the response variable. Missing data were treated as an additional state. After fitting the model with 50 SNPs, we performed stepwise backwards model selection using the function stepAIC in library MASS [49] in R [50], to drop SNPs that did not improve the fit of the model more than expected for additional parameters.

To generate approximate null expectations for the MAF distribution of candidate SNPs, linear regression, and relationships between MAF and effect size, we generated 20 randomized datasets in which data for three phenotypes (height, nodules in lower roots, and lower root occupancy by strain M249) were randomly assigned to accessions (leaving genotype data intact). For each randomized dataset, we repeated the TASSEL analysis and fit a multiple linear model using the 50 SNPs with lowest P values as explanatory variables. We calculated the mean and standard deviation of the adjusted proportion of variance explained from the 20 randomized datasets for each trait. For each randomized data set we also calculated the correlation between the effect size and MAF of the top 50 and 200 SNPs, and the number of SNPs found with MAF 2–5%, 5–10% and >10%. We caution that randomized data are approximate null expectations because, to the extent that the K matrix is used in the mixed-linear model analyses, the data are not fully exchangeable. Nevertheless, the q-q plots of actual to expected P-values (Figures S3, S4) reveal little evidence for shared demographic history that was not accounted for by the inclusion of K in the GWA analyses.

### In silico SNP platforms

To compare performance of GWAS with sequence data to reduced representation genotyping platforms, we generated 100 *in silico* platforms of 250 K regularly spaced SNPs. Each platform was designed using SNP data from a 26-accession, deeply-sequenced ascertainment panel [11]. From these data, a single SNP (MAF >0.10) was randomly selected from each of the 224,339 1-kb windows that harbored segregating sites and an additional 25,661 SNPs were randomly selected to generate a genotyping platform with 250 K SNPs. An average of 195 K SNPs per platform met the criteria of MAF >0.02 and assayed in ≥100 accessions and therefore used in analyses. *In silico* GWAS was conducted for three traits (height, nodules in lower root, and occupancy in lower root) by extracting genotype and *P*- values for SNPs on the *in silico* platform using the same methods used for the sequence data.

## Supporting Information

Figure S1 **Mean coverage for each of the 226 accessions included in the GWAS.**
(TIF)

Figure S2 **Histograms of accession means for each trait (along the diagonal).** Above diagonal are bivariate scatterplots for the 226 accessions means, the line in each plot is the linear correlation between traits. Below diagonal are correlation values between each pair of traits.
(TIF)

Figure S3 **Quantile-quantile (Q-Q) plots with and without K for height and nodules in upper roots.**
(TIF)

Figure S4 **Manhattan, quantile-quantile, and LD (top 50 SNPs) plots for all traits.**
(PDF)

Figure S5 **Minor allele frequency (MAF) distribution of all SNPs with MAF >0.02 and the top 200 candidates for each of the eight phenotypic traits.**
(TIF)

Figure S6 **Histograms of minor allele frequency (MAF, only SNPs with MAF >0.02 are included).** a) Sequence-based candidate SNPs, b) *in silico* candidate SNPs, and c) sequence-based candidate SNPs within 1 kb of *in silico* candidates for nodules in lower roots and strain occupancy in lower roots.
(TIFF)

Figure S7 **Neighbor-joining tree based on 5,000 randomly selected SNPs showing relatedness of all 288 sequenced accessions.** Trees constructed with other 5,000 SNP samples were qualitatively similar. The distinct clade shown in the middle of the tree represents the 18 accessions that were removed prior to analyses.
(PDF)

Table S1 **Results of GWAS conducted on 20 sets of randomized data for each of three traits (height, nodules in lower roots, and occupancy in lower roots).**
(DOCX)

Data File S1 **List of genomic location, annotation, p values, and expression for the top 200 candidates SNPs (those with lowest P values) for each of the eight phenotypes.**
(CSV)
