# Supplementary figures and images for: Candidate Genes and Genetic Architecture of Symbiotic and Agronomic Traits Revealed by Whole-Genome, Sequence-Based Association Genetics in Medicago truncatula
Source: PLoS One. 2013 May 31;8(5):e65688. doi: 10.1371/journal.pone.0065688 (PMC3669257; doi:10.1371/journal.pone.0065688)

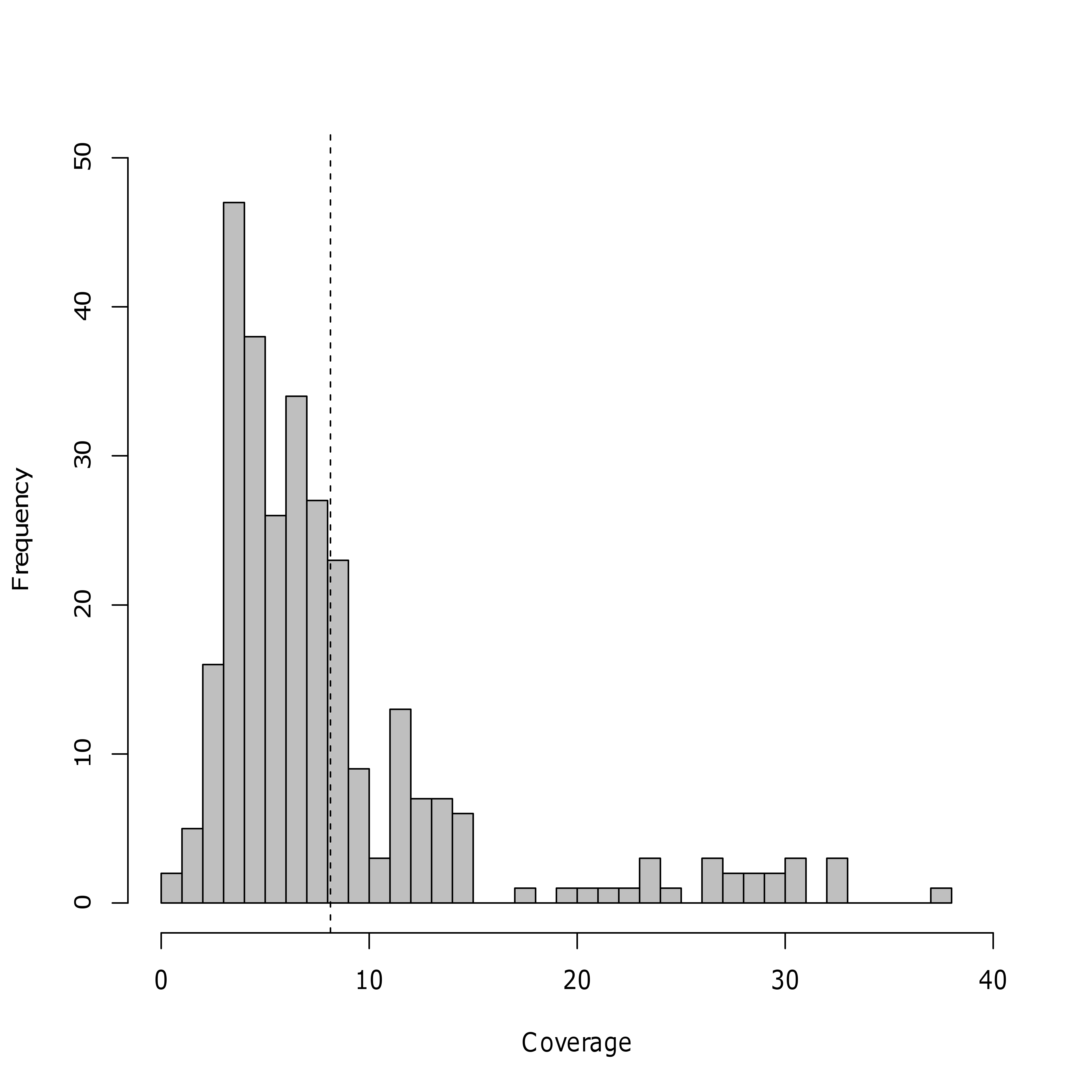

Supplement: Figure S1 — Mean coverage for each of the 226 accessions included in the GWAS. (TIF) [file pone.0065688.s001.tif]

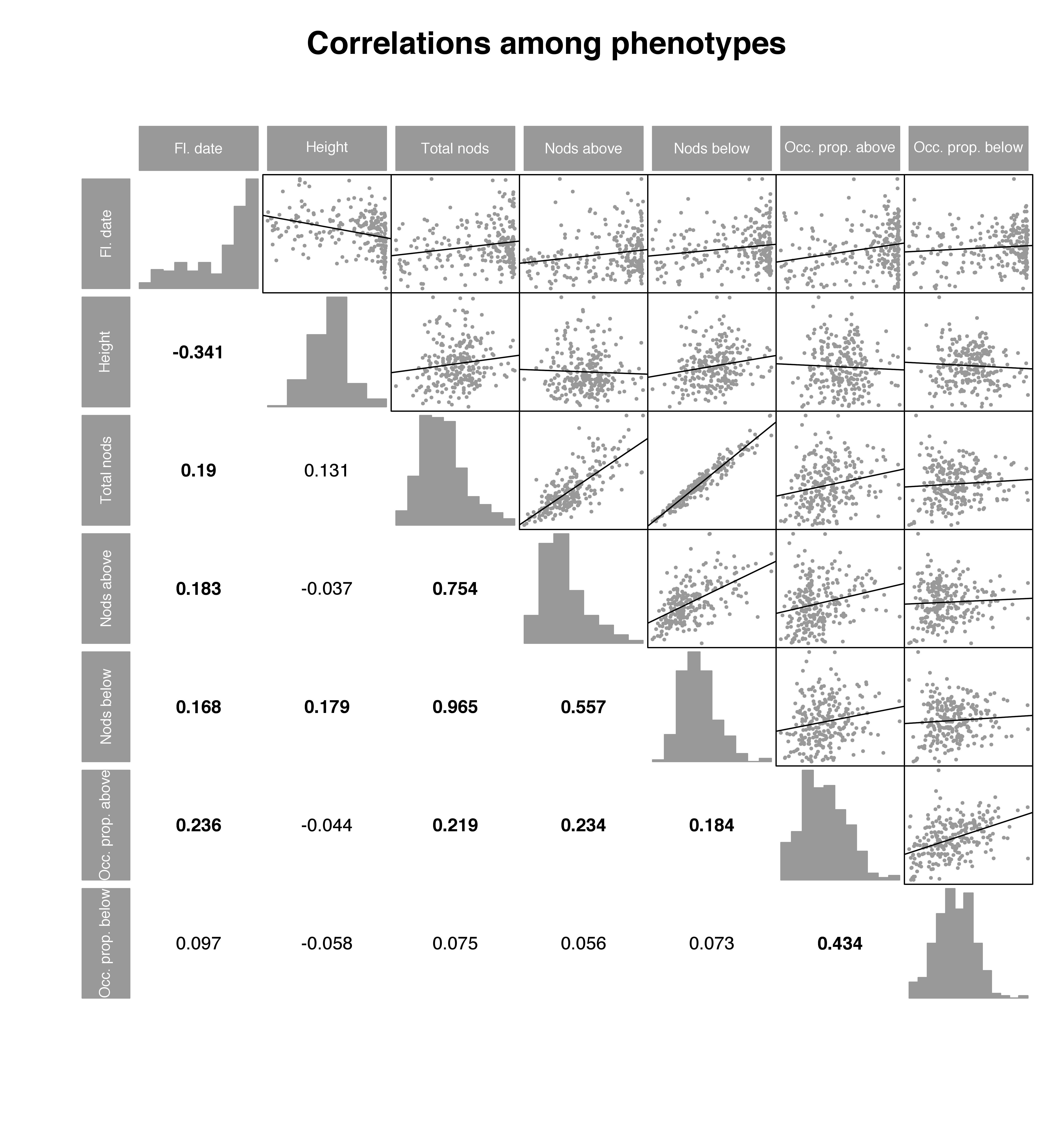

Supplement: Figure S2 — Histograms of accession means for each trait (along the diagonal). Above diagonal are bivariate scatterplots for the 226 accessions means, the line in each plot is the linear correlation between traits. Below diagonal are correlation values between each pair of traits. (TIF) [file pone.0065688.s002.tif]

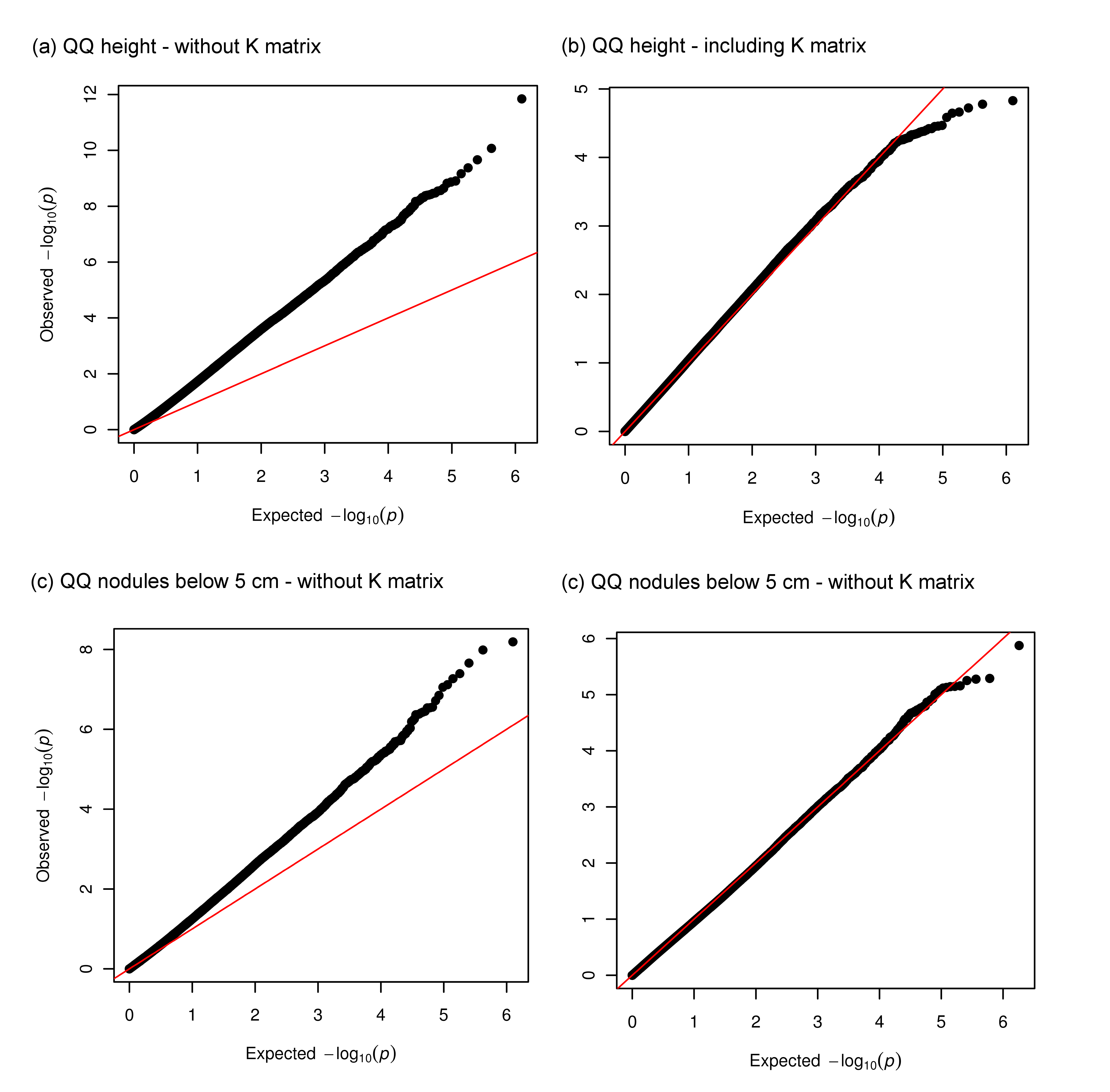

Supplement: Figure S3 — Quantile-quantile (Q-Q) plots with and without K for height and nodules in upper roots. (TIF) [file pone.0065688.s003.tif]

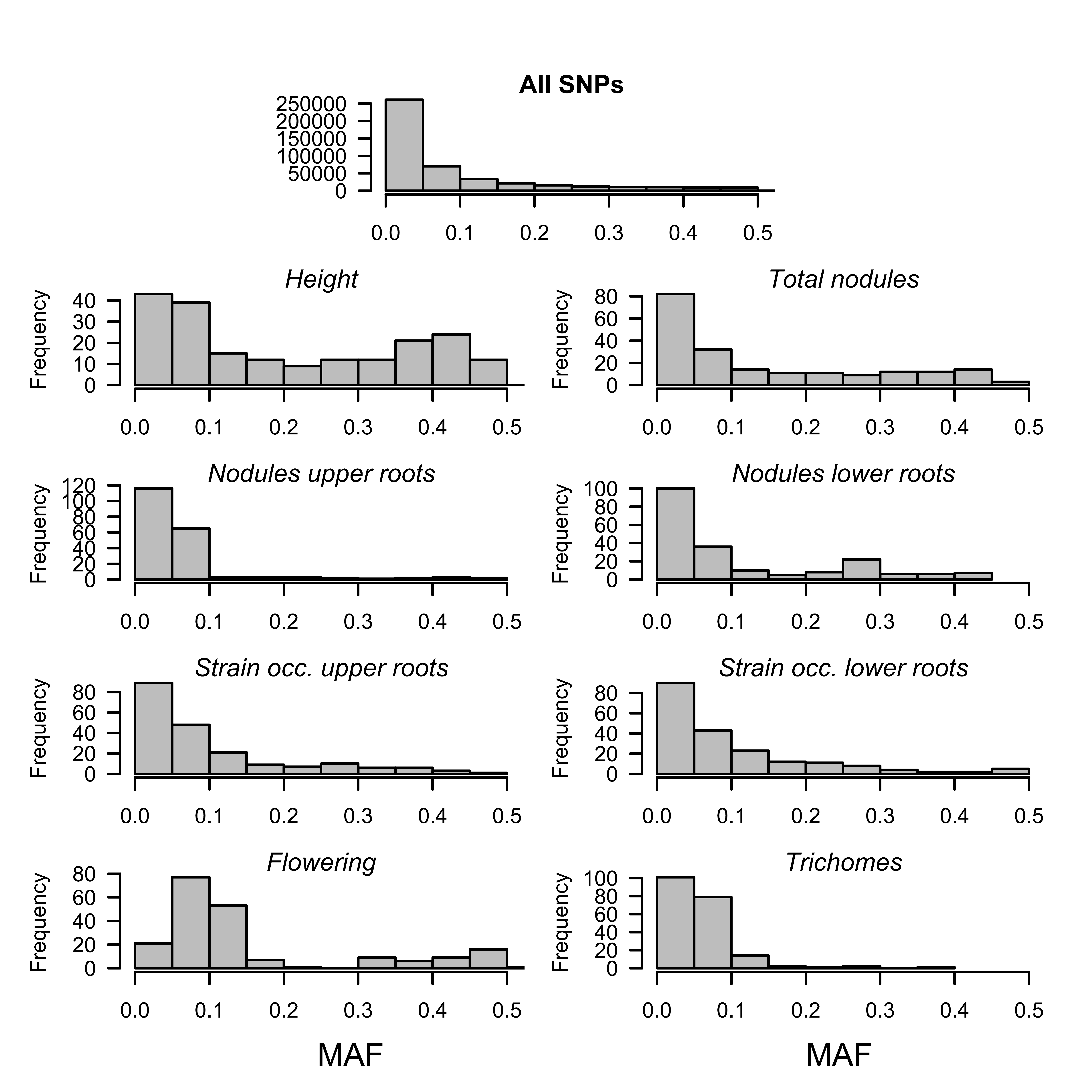

Supplement: Figure S5 — Minor allele frequency (MAF) distribution of all SNPs with MAF >0.02 and the top 200 candidates for each of the eight phenotypic traits. (TIF) [file pone.0065688.s005.tif]

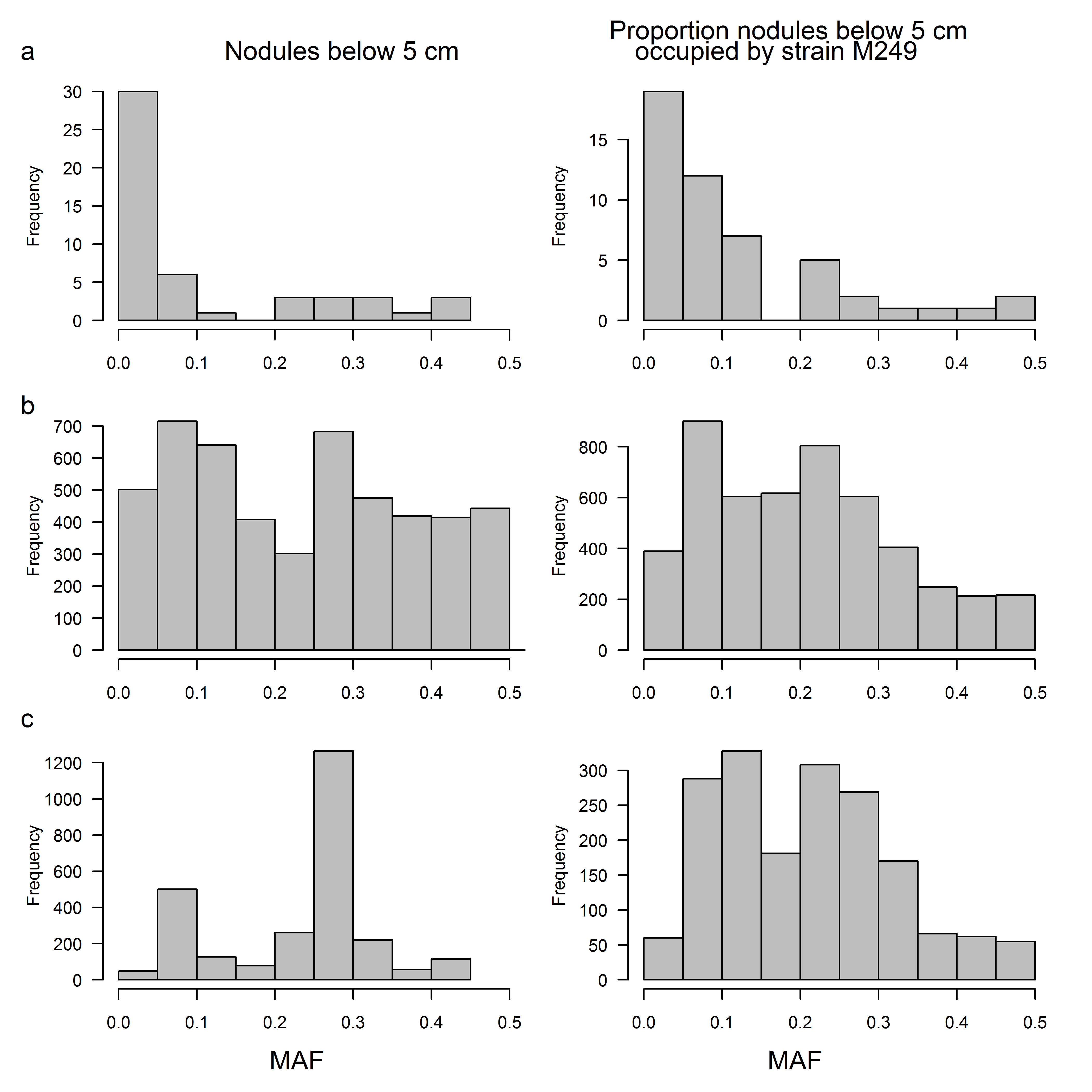

Supplement: Figure S6 — Histograms of minor allele frequency (MAF, only SNPs with MAF >0.02 are included). a) Sequence-based candidate SNPs, b) in silico candidate SNPs, and c) sequence-based candidate SNPs within 1 kb of in silico candidates for nodules in lower roots and strain occupancy in lower roots. (TIFF) [file pone.0065688.s006.tif]

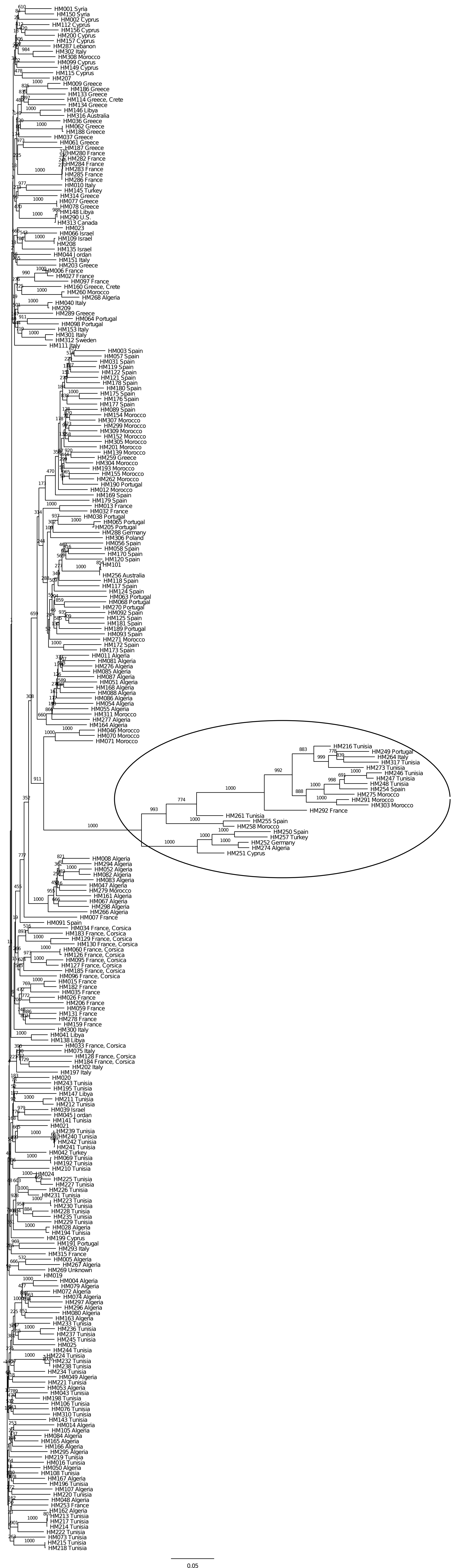

Supplement: Figure S7 — Neighbor-joining tree based on 5,000 randomly selected SNPs showing relatedness of all 288 sequenced accessions. Trees constructed with other 5,000 SNP samples were qualitatively similar. The distinct clade shown in the middle of the tree represents the 18 accessions that were removed prior to analyses. (PDF) [file pone.0065688.s007.pdf]
