# Supplementary material for: Candidate Genes and Genetic Architecture of Symbiotic and Agronomic Traits Revealed by Whole-Genome, Sequence-Based Association Genetics in Medicago truncatula
Source: PLoS One. 2013 May 31;8(5):e65688. doi: 10.1371/journal.pone.0065688 (PMC3669257; doi:10.1371/journal.pone.0065688)
Supplement: Figure S4 — Manhattan, quantile-quantile, and LD (top 50 SNPs) plots for all traits. (PDF) [file pone.0065688.s004.pdf]

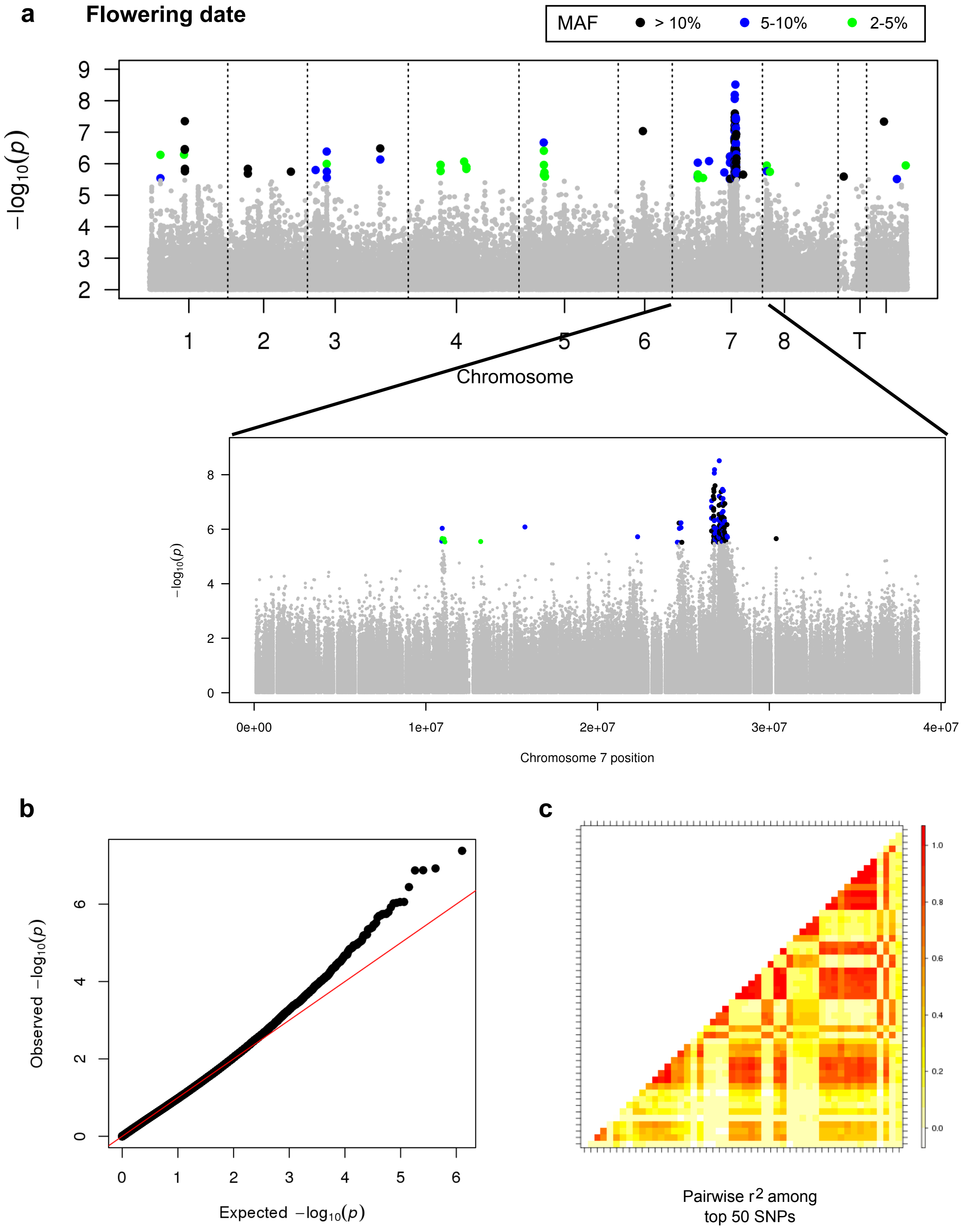
a
Flowering date
MAF
> 10%
5-10%
2-5%
−log10(p)
Chromosome
1
2
3
4
5
6
7
8
T
Chromosome 7 position
0e+00
1e+07
2e+07
3e+07
4e+07
b
Observed −log10(p)
Expected −log10(p)
c
Pairwise r2 among
top 50 SNPs
1.0
0.8
0.6
0.4
0.2
0.0

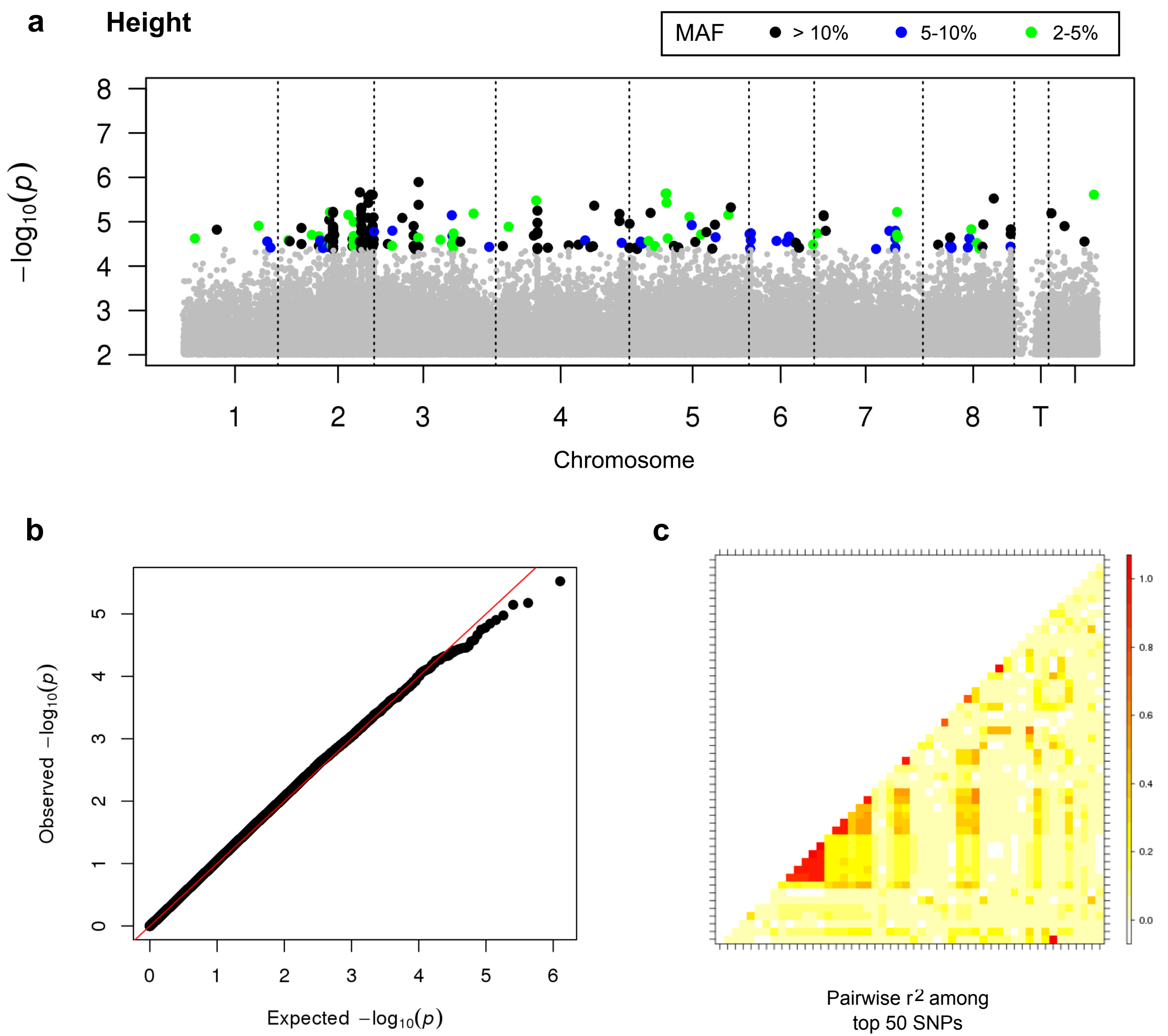

a
Height
MAF
> 10%
5-10%
2-5%
−log10(p)
Chromosome
1
2
3
4
5
6
7
8
T
b
Observed −log10(p)
Expected −log10(p)
c
Pairwise r2 among top 50 SNPs

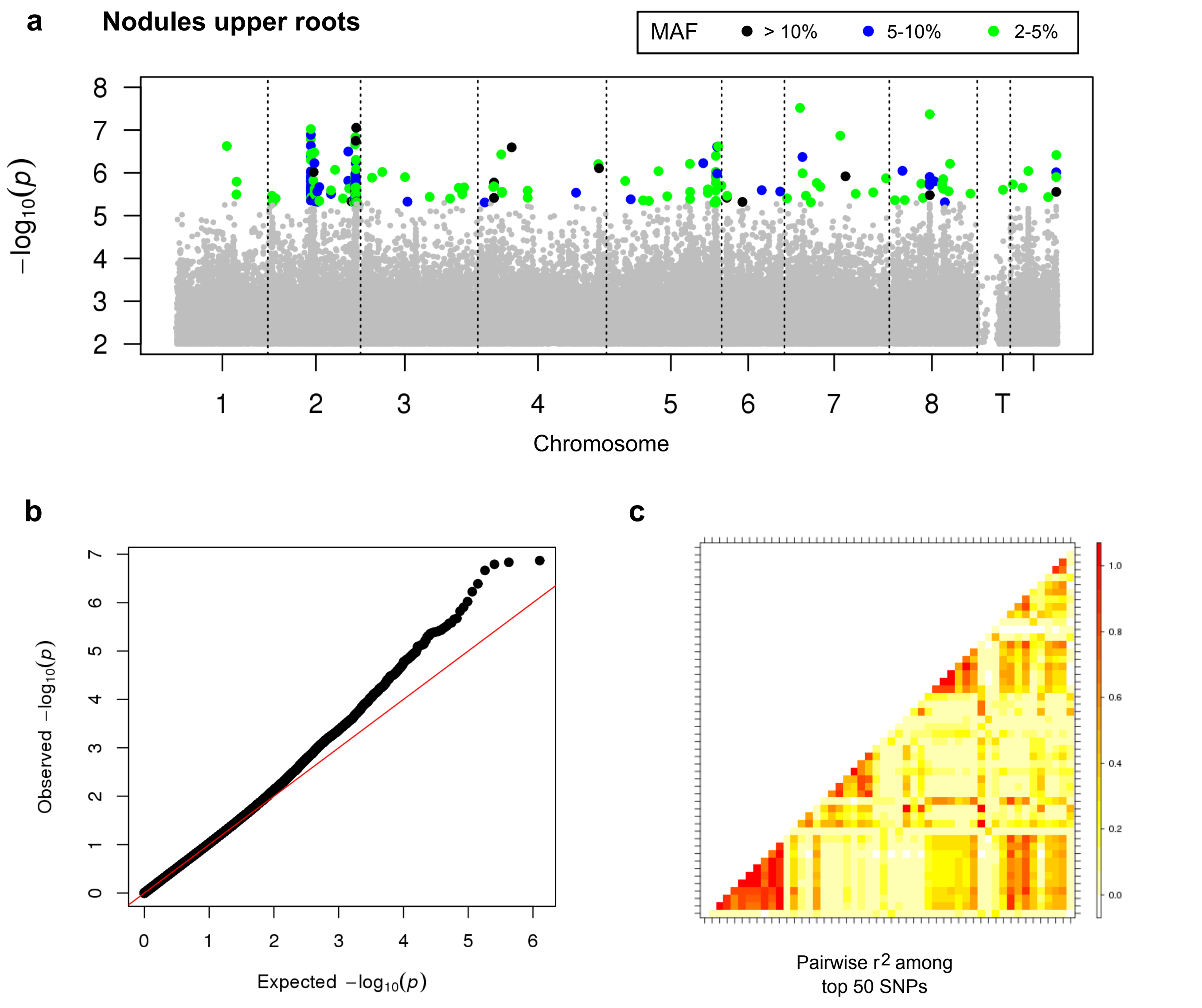

a
Nodules upper roots
MAF
> 10%
5-10%
2-5%
−log10(p)
Chromosome
1
2
3
4
5
6
7
8
T
b
Observed −log10(p)
Expected −log10(p)
c
Pairwise r2 among
top 50 SNPs
1.0
0.8
0.6
0.4
0.2
0.0

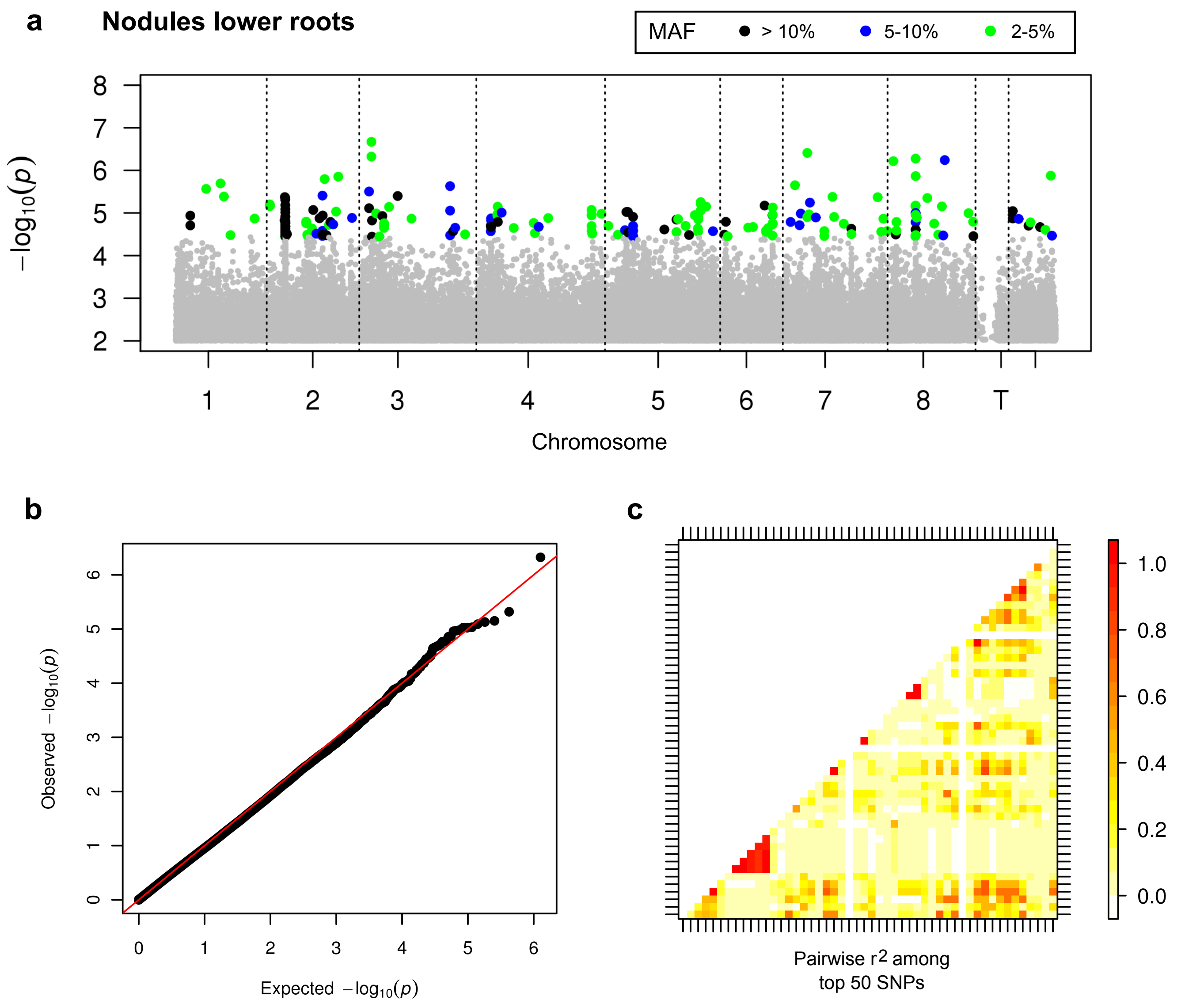

a
Nodules lower roots
MAF
> 10%
5-10%
2-5%
−log10(p)
Chromosome
1
2
3
4
5
6
7
8
T
b
Observed −log10(p)
Expected −log10(p)
c
Pairwise r2 among
top 50 SNPs

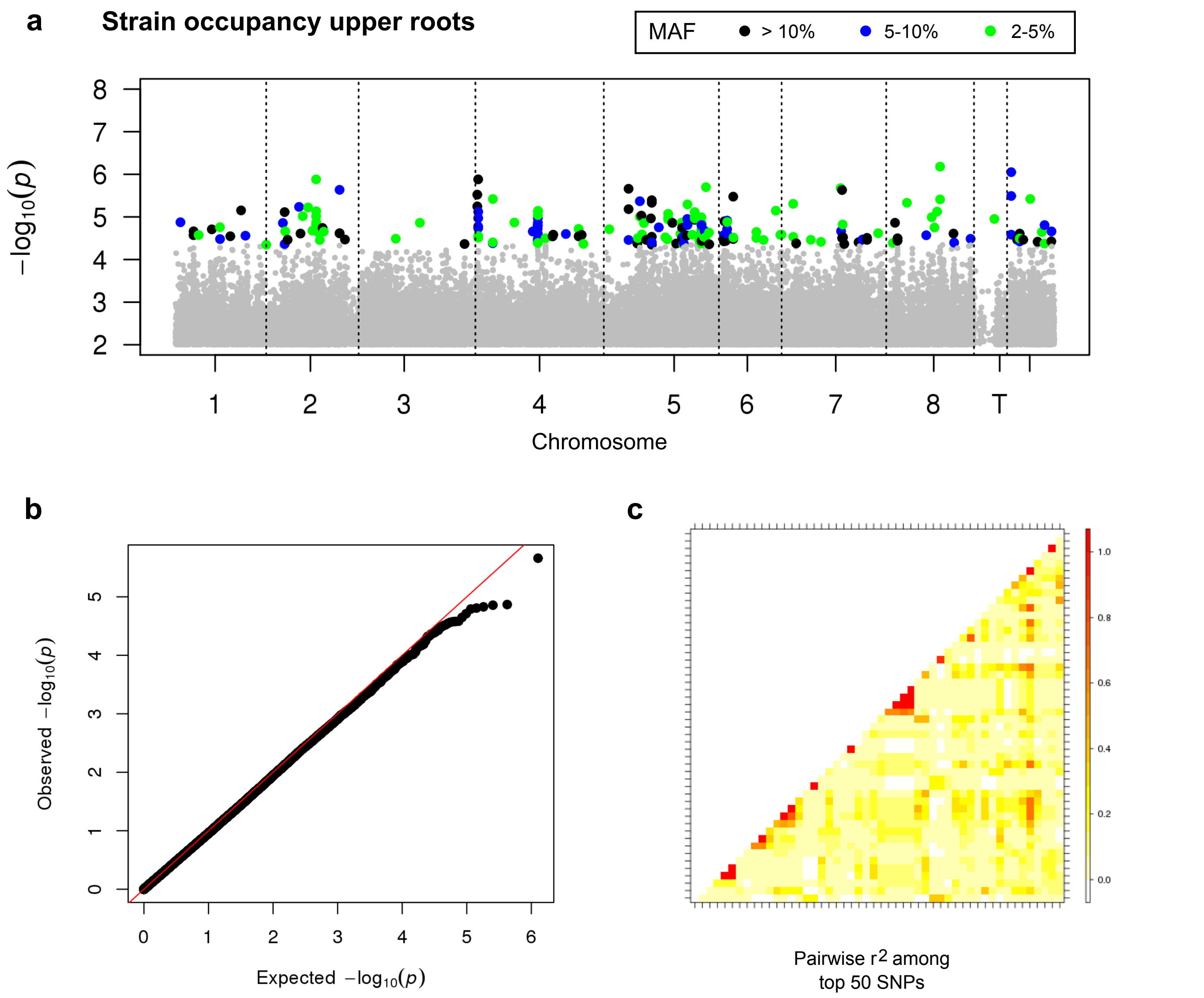
a
Strain occupancy upper roots
MAF
> 10%
5-10%
2-5%
−log10(p)
Chromosome
1
2
3
4
5
6
7
8
T
b
Observed −log10(p)
Expected −log10(p)
c
Pairwise r2 among
top 50 SNPs

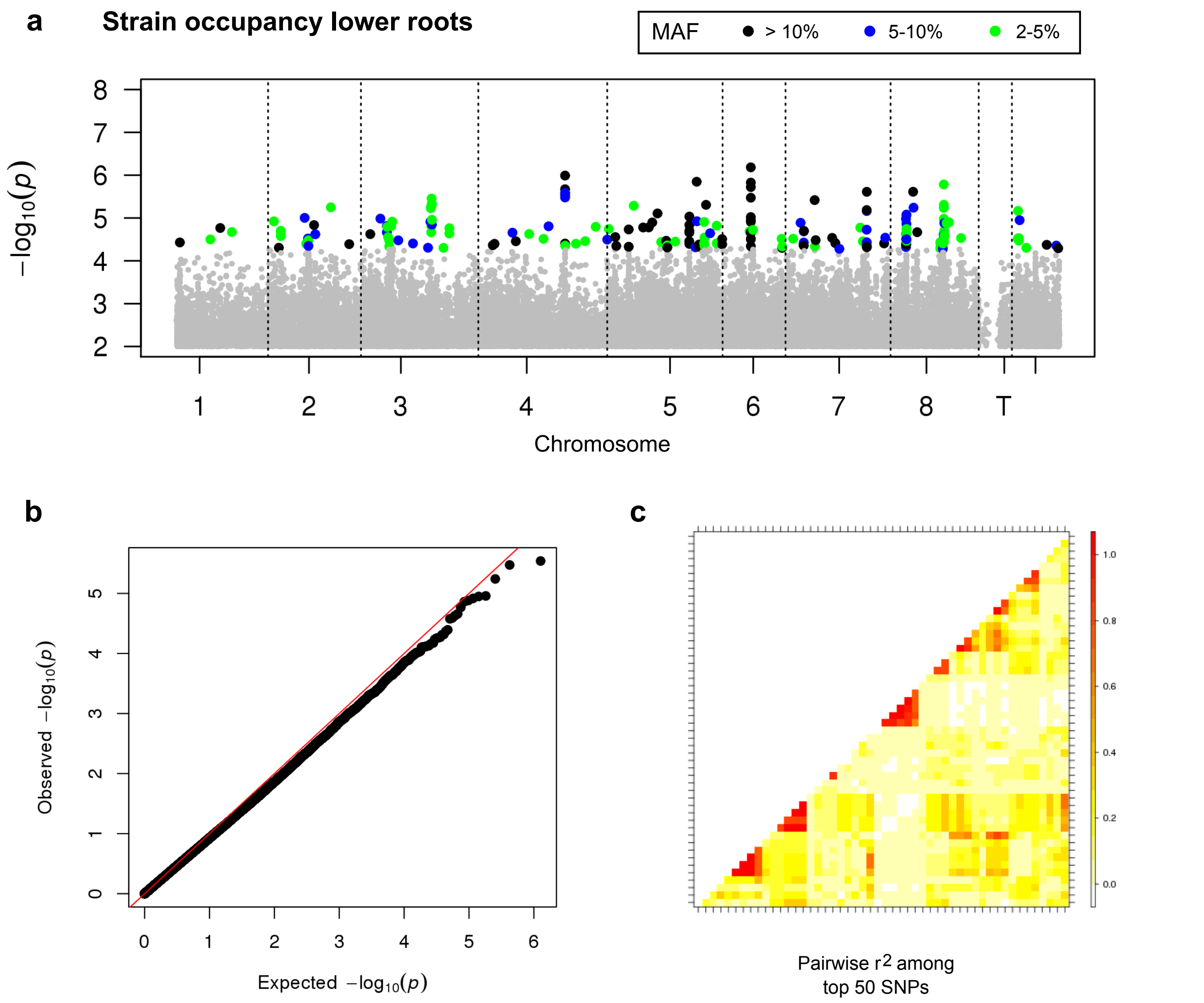
a
Strain occupancy lower roots
MAF
> 10%
5-10%
2-5%
−log10(p)
8
7
6
5
4
3
2
1
2
3
4
5
6
7
8
T
Chromosome
b
Observed −log10(p)
Expected −log10(p)
0
1
2
3
4
5
6
c
1.0
0.8
0.6
0.4
0.2
0.0
Pairwise r2 among
top 50 SNPs

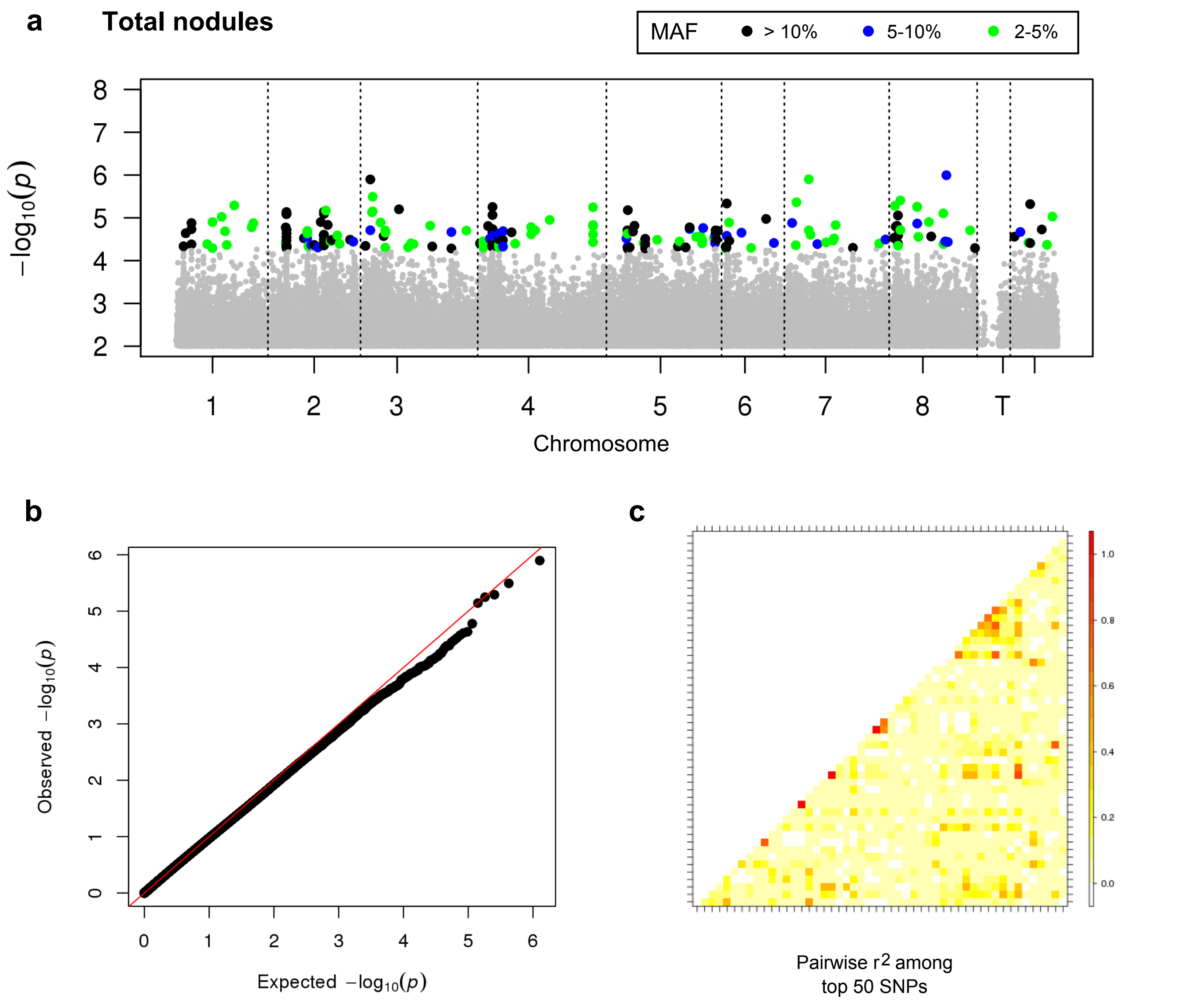
a
Total nodules
MAF
> 10%
5-10%
2-5%
−log10(p)
Chromosome
1
2
3
4
5
6
7
8
T
b
Observed −log10(p)
Expected −log10(p)
c
Pairwise r2 among
top 50 SNPs

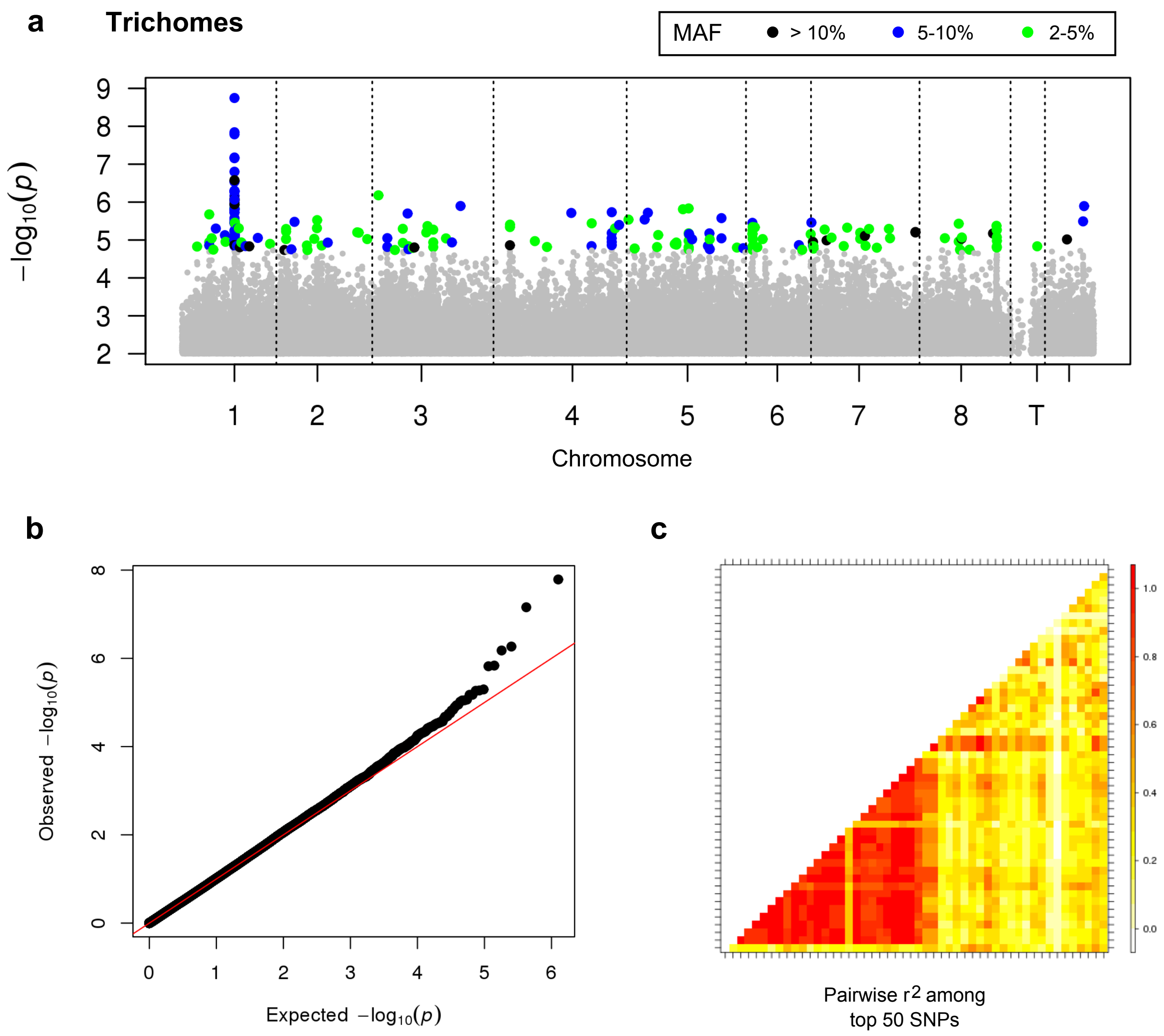
a
Trichomes
MAF
> 10%
5-10%
2-5%
−log10(p)
Chromosome
1
2
3
4
5
6
7
8
T
b
Observed −log10(p)
Expected −log10(p)
c
Pairwise r2 among
top 50 SNPs
