# Supplementary material for: Candidate Genes and Genetic Architecture of Symbiotic and Agronomic Traits Revealed by Whole-Genome, Sequence-Based Association Genetics in Medicago truncatula
Source: PLoS One. 2013 May 31;8(5):e65688. doi: 10.1371/journal.pone.0065688 (PMC3669257; doi:10.1371/journal.pone.0065688)
Supplement: Table S1 — Results of GWAS conducted on 20 sets of randomized data for each of three traits (height, nodules in lower roots, and occupancy in lower roots). (DOCX) [file pone.0065688.s008.docx]

Table S1. Results of GWAS conducted on 20 sets of randomized data for each of three traits (height, nodules in lower roots, and occupancy in lower roots). MAF distribution of top 50 SNPs, adjusted proportion of variance explained in a linear model with top 50 candidate SNPs as explanatory variables, and the correlation (*r)* between MAF and effect size for top 50 and top 200 candidate SNPs. Permutations were performed by randomly assigning phenotypic values to accessions, leaving genotypic values intact. Empirical values shown on bottom row are values obtained from the actual data.

| Trait | Permutation | # SNPs MAF 2-5% | # SNPs MAF 5-10% | # SNPs MAF > 10% | Adjusted r^2^ (SNPs in final model) | *r* MAF – effect size  top 200 SNPs | *r* MAF – effect size  top 50 SNPs |
| --- | --- | --- | --- | --- | --- | --- | --- |
| Height | 1 | 21 | 15 | 14 | 0.69 (34) | -0.08 | -0.20 |
|  | 2 | 34 | 6 | 10 | 0.63 (31) | 0.32 | 0.30 |
|  | 3 | 34 | 8 | 8 | 0.54 (24) | 0.12 | 0.29 |
|  | 4 | 29 | 11 | 10 | 0.67 (24) | 0.03 | -0.16 |
|  | 5 | 24 | 13 | 13 | 0.67 (35) | 0.20 | 0.19 |
|  | 6 | 27 | 2 | 21 | 0.70 (26) | -0.08 | -0.23 |
|  | 7 | 30 | 8 | 12 | 0.58 (30) | -0.10 | -0.32 |
|  | 8 | 37 | 6 | 7 | 0.65 (23) | 0.01 | 0.08 |
|  | 9 | 27 | 13 | 10 | 0.68 (31) | 0.16 | 0.19 |
|  | 10 | 24 | 14 | 12 | 0.62 (23) | 0.29 | -0.06 |
|  | 11 | 24 | 16 | 10 | 0.70 (29) | 0.12 | 0.29 |
|  | 12 | 33 | 3 | 14 | 0.69 (27) | 0.29 | 0.48 |
|  | 13 | 28 | 10 | 12 | 0.66 (28) | 0.01 | -0.01 |
|  | 14 | 19 | 10 | 21 | 0.66 (24) | -0.09 | -0.03 |
|  | 15 | 27 | 9 | 14 | 0.68 (26) | 0.07 | 0.27 |
|  | 16 | 35 | 7 | 8 | 0.62 (30) | 0.28 | 0.35 |
|  | 17 | 35 | 9 | 6 | 0.70 (23) | 0.09 | 0.06 |
|  | 18 | 27 | 9 | 14 | 0.68 (29) | 0.03 | -0.23 |
|  | 19 | 22 | 18 | 10 | 0.51 (25) | 0.08 | 0.17 |
|  | 20 | 35 | 12 | 3 | 0.61 (31) | 0.11 | -0.16 |
|  | mean | 28.6 | 10.0 | 11.5 | 0.65 | 0.09 | 0.06 |
| *empirical* |  | *13* | *2* | *35* | *0.75* | *-0.12* | *-0.35* |

| Trait | Permutation | # SNPs MAF 2-5% | # SNPs MAF 5-10% | # SNPs MAF > 10% | Adjusted r^2^ (SNPs in final model) | *r* MAF – effect size  top 200 SNPs | *r* MAF – effect size  top 50 SNPs |
| --- | --- | --- | --- | --- | --- | --- | --- |
| Nod B | 1 | 34 | 8 | 8 | 0.58 (28) | 0.11 | 0.20 |
|  | 2 | 40 | 9 | 1 | 0.58 (27) | 0.01 | -0.02 |
|  | 3 | 31 | 11 | 8 | 0.54 (20) | -0.09 | 0.17 |
|  | 4 | 41 | 4 | 5 | 0.63 (32) | 0.11 | 0.16 |
|  | 5 | 31 | 11 | 8 | 0.72 (29) | 0.03 | 0.08 |
|  | 6 | 36 | 8 | 6 | 0.57 (21) | 0.16 | 0.22 |
|  | 7 | 41 | 7 | 2 | 0.52 (22) | 0.12 | -0.15 |
|  | 8 | 37 | 9 | 4 | 0.65 (29) | 0.02 | -0.03 |
|  | 9 | 31 | 10 | 9 | 0.65 (30) | 0.02 | -0.05 |
|  | 10 | 37 | 12 | 1 | 0.65 (32) | -0.06 | -0.14 |
|  | 11 | 39 | 7 | 4 | 0.52 (26) | 0.30 | 0.42 |
|  | 12 | 39 | 9 | 2 | 0.61 (26) | 0.09 | 0.26 |
|  | 13 | 31 | 9 | 10 | 0.59 (24) | -0.12 | -0.38 |
|  | 14 | 45 | 4 | 1 | 0.59 (22) | 0.12 | 0.00 |
|  | 15 | 37 | 5 | 8 | 0.56 (25) | 0.04 | -0.30 |
|  | 16 | 34 | 11 | 5 | 0.62 (27) | 0.09 | 0.18 |
|  | 17 | 35 | 13 | 2 | 0.62 (22) | -0.02 | 0.12 |
|  | 18 | 30 | 17 | 3 | 0.64 (29) | -0.03 | -0.15 |
|  | 19 | 39 | 8 | 3 | 0.63 (32) | 0.14 | 0.33 |
|  | 20 | 33 | 11 | 6 | 0.67 (28) | 0.11 | 0.10 |
|  | average | 36.1 | 9.2 | 4.8 | 0.61 | 0.05 | 0.05 |
| *empirical* |  | *30* | *6* | *14* | *0.69* | *-0.21* | *-0.25* |

| Trait | Permutation | # SNPs MAF 2-5% | # SNPs MAF 5-10% | # SNPs MAF > 10% | Adjusted r^2^ (SNPs in final model) | *r* MAF – effect size  top 200 SNPs | *r* MAF – effect size  top 50 SNPs |
| --- | --- | --- | --- | --- | --- | --- | --- |
| Nodule | 1 | 34 | 11 | 5 | 0.56 (25) | 0.07 | -0.01 |
| occupancy | 2 | 39 | 5 | 6 | 0.53 (21) | 0.15 | 0.26 |
| by strain | 3 | 28 | 12 | 10 | 0.59 (25) | 0.15 | 0.25 |
| M249 | 4 | 36 | 6 | 8 | 0.62 (31) | 0.06 | -0.17 |
| below | 5 | 32 | 14 | 4 | 0.66 (35) | 0.02 | 0.06 |
| 5 cm | 6 | 35 | 6 | 9 | 0.63 (25) | 0.07 | 0.24 |
|  | 7 | 44 | 4 | 2 | 0.41 (20) | 0.14 | 0.62 |
|  | 8 | 36 | 11 | 3 | 0.56 (22) | 0.14 | 0.21 |
|  | 9 | 32 | 14 | 4 | 0.52 (26) | -0.01 | 0.05 |
|  | 10 | 35 | 3 | 12 | 0.71 (33) | 0.22 | 0.03 |
|  | 11 | 40 | 4 | 6 | 0.75 (29) | 0.20 | 0.48 |
|  | 12 | 14 | 6 | 30 | 0.53 (22) | 0.09 | 0.14 |
|  | 13 | 34 | 12 | 4 | 0.65 (27) | 0.11 | 0.15 |
|  | 14 | 28 | 12 | 10 | 0.64 (25) | 0.12 | 0.10 |
|  | 15 | 26 | 12 | 12 | 0.56 (22) | 0.02 | 0.21 |
|  | 16 | 22 | 22 | 6 | 0.59 (31) | 0.21 | 0.27 |
|  | 17 | 29 | 14 | 7 | 0.63 (28) | 0.25 | 0.27 |
|  | 18 | 30 | 13 | 7 | 0.61 (29) | 0.14 | 0.20 |
|  | 19 | 37 | 6 | 7 | 0.54 (21) | 0.29 | 0.59 |
|  | 20 | 38 | 7 | 5 | 0.62 (31) | 0.20 | 0.01 |
|  | average | 32.5 | 9.7 | 7.9 | 0.59 (26) | 0.13 | 0.20 |
| *empirical* |  | *19* | *12* | *19* | *0.61* | *0.17* | *0.22* |
